# Implementation of a Novel Wilderness Medicine Simulation Course for Medical Students

**DOI:** 10.15766/mep_2374-8265.11526

**Published:** 2025-06-09

**Authors:** Katherine A. Sprengel, Sophia Redpath, Kira L. Palazzo, Sara M. Hock

**Affiliations:** 1 Third-Year Medical Student, Rush Medical College of Rush University Medical Center; 2 Associate Professor and Director of Simulation, Department of Emergency Medicine, Rush Medical College of Rush University Medical Center

**Keywords:** Wilderness Medicine, Simulation, Case-Based Learning, Emergency Medicine, Pediatric Emergency Medicine

## Abstract

**Introduction:**

Wilderness medicine is a growing field focused on delivering quality medical care in austere environments. Simulation-based education has proven effective in emergency and wilderness medicine, particularly in graduate medical education. We propose that introducing wilderness medicine concepts earlier in medical education as part of a high-fidelity simulation for medical students could both increase interest in wilderness medicine and have widely applicable educational benefits.

**Methods:**

We developed a novel 1-day case-based simulation curriculum to be performed in a wilderness environment and invited undergraduate medical students to participate. A 25-question survey was administered before and after the simulation to assess subjective change across various topics.

**Results:**

The 10 of 12 students who responded to the survey indicated that the simulation significantly increased their confidence in managing urgent medical cases and increased their interest in the wilderness medicine field. All students agreed that simulation was an effective way to learn this material.

**Discussion:**

Implementing a wilderness medicine simulation in medical curricula appears feasible and provides a comprehensive model that can be easily adapted to other institutions.

## Educational Objectives

By the end of this activity, learners will be able to:

1. Demonstrate the proper approach to a medical emergency in a wilderness setting, including considerations for environmental safety, team communication, and procedures for patient evacuation.
2. Intervene with appropriate medical care in the following five cases: anaphylaxis, splinting, hypothermia and concussion, choking child, and arterial bleed.
3. Discuss key elements of the field of wilderness medicine.

## Introduction

Wilderness medicine (hereafter WM) entails the application of clinical medicine in an uncontrolled, resource-limited environment. Much like the field of emergency medicine, of which WM is a branch, it requires a wide breadth of medical knowledge and skills, quick thinking, and effective communication and teamwork. WM is a quickly growing field: in 2013, there were 12 institutions offering WM fellowships,^1^ while as of 2023 there are 22 programs in the field.^2^

Congruent with growing interest, the use of simulation has been gaining traction in the field of WM education. Due to the environmental requirements of in situ wilderness medical training, it is a notoriously difficult field for medical trainees to access. Incorporating a simulation-based curriculum can help bridge that gap. The potential benefits of simulation include experiential learning, immediate feedback, hands-on practice, and active decision-making.^3,4^ Simulation allows students to practice in a low-stakes environment, which may decrease anxiety and increase confidence, better preparing them for learning in clinical settings with real patients.^5^ In fact, there is evidence to suggest that the use of simulation in both trainee and provider education may improve patient outcomes.^6,7^ Previous reports have described the use of simulation specifically for WM education; however, these activities have been primarily aimed at graduate medical students in emergency medicine or WM-specific programs.^8–13^ Prior educational models have ranged from low-fidelity simulation to practice procedural skills,^10^ to a high-fidelity, repeatedly implemented pediatric case.^9^ These cases provide valuable educational opportunities in the field; however, medical students are notably absent from the intended learner demographics. Given the importance of WM and the growing field of graduate WM education, there is a need to broaden the scope of undergraduate education and introduce the field earlier in the time line of medical education. We aimed to fill this gap in simulation-based WM education.

Interestingly, students who participated in some form of WM curriculum during their education report higher levels of resilience and critical thinking.^10^ Foundational elements of simulation-based WM training, such as critical thinking and improvisation, can be widely applicable to general undergraduate medical training. Moreover, WM electives for fourth-year medical students have been shown to increase student interest in pursuing further WM involvement after medical school.^14^ As such, we hypothesize that introducing WM education to preclinical medical students will both positively affect their education as a whole and foster interest in the field, and may influence their future career paths.

We report on the development and implementation of a WM simulation curriculum aimed at first-, second-, and third-year medical students. We also report on the development and use of five cases that could be used and adapted to other educational WM simulation models.

## Methods

### Development

We followed Kern's 6-step model^15^ for curricular development by first performing a needs assessment focused on the learning needs of novices in the field, with the aim of providing an educational experience that is accessible but also presents some novel challenges. Developers of the curriculum included the authors (Katherine A. Sprengel, Sophia Redpath, Kira L. Palazzo, Sara M. Hock).

We developed five cases (Appendices A–E) to address selected objectives of environmental exposure, traumatic injury intervention, and medical management in a wilderness setting. Low-fidelity simulation was selected as the educational strategy, with optional preparatory materials and a postsimulation debrief to deliver the curricular content.

The curriculum was implemented in a local forest preserve and used a variety of low-fidelity simulation equipment (i.e., manikin, active bleeding arms, moulage) to increase the authenticity. Given the challenge of transporting manikin trainers, our team elected to use live actors in moulage with appropriate task trainers as patients for the simulation. For the purposes of curriculum evaluation, we developed pre- and postsimulation surveys (Appendix F) to facilitate the feedback portion of curricular development. The curriculum was reviewed and determined to be exempt by the Rush University Medical Center Institutional Review Board.

The intended audience of novice medical student learners was required to perform an appropriately conservative level of medical intervention for this workshop. We (Katherine A. Sprengel, Sophia Redpath, Kira L. Palazzo, Sara M. Hock) created the five cases for the workshop to represent common injuries and medical problems that may be encountered in the wilderness. The overall simplicity of these cases was intended to allow the participants, most of whom were previously unfamiliar with WM, to focus their learning on the challenges and adaptations required by the limiting environment, rather than on more complicated medical interventions. Future users of this curriculum are encouraged to tailor the complexity of cases to meet the educational level of their participants.

### Implementation

Prior to the course, the team obtained permission from the park district to host this event. We chose a loop trail and evenly spaced each case scenario along it. Equipment and personnel requirements for the setup of each case are listed below; further details are included in Appendices A–E. Each participant in this curriculum wore a colored pinnie to indicate participation in a learning course. There were signs posted to indicate to passersby that this was for educational purposes and there was no real danger. Any further iteration of this curriculum taking place in a public setting must also consider the safety of nonparticipants. Participant safety was also considered, with encouragement to bring appropriate footwear, clothing, hydration, and snacks.

When students arrived at the forest preserve, they were divided into groups of three to four participants per group. Each group was given a backpack containing a variety of medical equipment (details on backpack contents listed below). Participants gathered for a prebriefing session that included an overview of what to expect for the day, instructions on how to use the provided equipment, and an introduction to WM skills—for example, key requirements for improvising a splint (Appendix G). Participants were instructed to use the equipment at their discretion according to each case they encountered.

Each group started at a different site along the trail, and all started their case at the same time. Each case site was given an overall time limit of 20 minutes. The average time for execution of cases was 5 minutes, leaving the majority of the time at each station for debrief and education. Upon completion of each case and the corresponding debrief, groups moved clockwise by walking around the loop trail until they encountered the next scenario. The groups were spaced approximately 5–10 minutes apart. Leadership communicated via text to ensure groups traveled between sites with coordinated timing. A full description of the intended flow and key learning points from each case can be found in Appendices A–E.

The following are key teaching points specific to the wilderness aspect of this curriculum. These skills are mentioned in the prebriefing material and in the case descriptions but should also be thoroughly discussed during the debriefing.

#### Anaphylaxis (Case 1; Appendix A)

Even in wilderness settings, anaphylaxis is most effectively treated by an epinephrine autoinjector (EpiPen) that is carried by the person with the allergy. Many first aid kits may include diphenhydramine or other antihistamines; however, this case emphasizes the importance of abrupt recognition and prompt intervention, followed by expeditious evacuation for further care.

#### Trimalleolar fracture (Case 2; Appendix B)

While participants had access to a structural aluminum malleable (SAM) splint and all cotton elastic (ACE) bandages, it was emphasized that WM often necessitates improvisation without use of commercial medical equipment. Educators reiterated the key elements of a splint as outlined in the prebriefing materials, and learners were encouraged to practice utilizing nonmedical materials. Splinting should utilize a rigid object, like a stick or rolled item of clothing, to immobilize the extremity both above and below the fractured joint while using some type of fastening material, like a backpack strap or piece of fabric, to secure it.

#### Traumatic bleeding control (Case 3; Appendix C)

Participants in our course had access to a tourniquet and practiced applying it correctly. This included placing the tourniquet 2–3 inches proximal to the wound, avoiding joints, and tightening until hemostasis is achieved. Subsequent discussion focused on how to improvise a tourniquet, which emphasized the role of the windlass (i.e., a firm stick) in providing the force applied by the band (i.e., a piece of clothing or strap) required for hemostasis to be achieved. In fact, there are some data showing that an improvised windlass can actually be nearly, if not equally, as effective as a commercially produced tourniquet if used correctly.^16^

#### Hypothermia (Case 4; Appendix D)

Many considerations in caring for a patient with hypothermia in the wilderness were presented. For example, participants were instructed to take note of the wet clothes on the patient and replace them with warm, dry clothing. A discussion of additional key elements included the following: a physical barrier between the patient and the ground, active rewarming techniques, nutritional support, and recognition of decompensation.

### Equipment and Personnel

The personnel and equipment required for each case, along with a brief description of the initial appearance of the simulated patient, are as follows:

#### Anaphylaxis (Case 1; Appendix A)

For this scenario, one manikin or simulation leader is needed to play the patient, and one physician simulation leader is required to oversee the scenario and run the debrief. The equipment includes a granola bar wrapper or another visual indicator of a common allergen, as well as a practice EpiPen in the participant's backpack. The patient will start the case standing to the side of the trail, wearing a hiking backpack and holding the wrapper or other allergen indicator, with no moulage.

#### Trimalleolar fracture (Case 2; Appendix B)

One lower-extremity manikin or simulation leader is needed to play the patient, and one physician simulation leader is required to oversee the scenario and conduct the debrief. Equipment includes SAM splints, which are included in the participant's backpack. The patient will begin the case sitting on the trail, wearing hiking boots and long pants. No moulage will be used, but the instructor will provide a verbal description of the deformity.

#### Traumatic bleeding control (Case 3; Appendix C)

In this scenario, one simulation leader will play the patient, with all interventions performed on a bleeding arm trainer (an upper-extremity manikin with arterial bleeding capability and a fluid reservoir connected to the arm). A physician simulation leader will oversee the scenario and run the debrief. Equipment includes the bleeding arm trainer, as well as a tourniquet and gauze that are included in the participant's backpack. The patient will be positioned lying on the side of the trail near a sharp rock or stick (or any other plausible object that could cause such a wound). Moulage will be applied to the wound on the simulated patient's arm.

#### Hypothermia (Case 4; Appendix D)

For this case, one full-body manikin or simulation leader is needed to play the patient, and one physician simulation leader will oversee the scenario and run the debrief. The equipment includes moulage to simulate a laceration with surrounding ecchymosis on the patient's forehead. The patient will begin lying down on the side of the trail, and if possible, near a body of water. The moulage will be applied, and the patient's clothing may be wet to reflect hypothermic conditions.

#### Choking child (Case 5; Appendix E)

This case requires one child manikin to simulate the patient and one physician simulation leader to oversee the scenario and run the debrief. If available, an additional simulation leader will play the role of the parent. The equipment includes a hiking backpack (or, alternatively, the patient can be held by the parent), as well as grapes or another small food item that is considered of sufficient size to cause a child to choke. The patient will start the case in the parent's hiking backpack or in the parent's arms, with the parent walking along the trail and holding the small food item. No moulage will be used in this scenario.

#### Contents of backpacks

Five backpacks in total were prepared, one per group of students. The backback contained the following items:

• Practice EpiPen injectors (1)
• SAM splints (1)
• Pinnies (4)
• Tourniquets (1)
• Trauma shears (1)
• Gauze (2)
• ACE wraps (1)
• Triangle bandages (1)

### Debriefing

Debriefing sessions were held at each site following completion of the case, led by emergency medicine physicians experienced in simulation-based medical education. Faculty utilized typical debriefing formats and provided key learning points (discussed above in Implementation) to share with students. Participants were encouraged to reflect on what they learned throughout the day, including specific medical knowledge as well as broader reflections on their communication styles and ability to adapt to a wilderness setting. Because our institution has a robust simulation program, our facilitators had experience in leading similar sessions; when this curriculum is applied at institutions without such a background, it is recommended that facilitators undergo training on the significance of debriefing and the suggested structure for conducting sessions. The specifics of this training are beyond the scope of this project.

### Assessment

Each simulation case was based on common themes of WM management, with a diverse range of specific medical management skills. The critical actions determined for each case are discussed in Appendix H; case-specific critical actions are embedded within each case. The week prior to the simulation, participants were asked to complete a presimulation survey to indicate their baseline experience with WM, and to rate their baseline levels of comfort in approaching wilderness medical emergencies and confidence in adapting medical knowledge to unconventional situations (each rated on a 1–10-point scale (1 = *lowest possible degree*, 10 = *highest possible degree*). Following the end of the simulation day, students completed a similar postsimulation survey that aimed to assess changes in the aforementioned parameters, as well as their overall perception of the effectiveness of simulation in WM education. The pre- and postsimulation surveys were sent via email and were completed anonymously by participants. Paired two-tailed *t* tests were applied to compare participant responses pre- and postsimulation and determine whether the intervention affected a variety of self-reported measures.

## Results

Overall, the WM Simulation Day was a success. In total, 12 medical students—10 M1s and two M2s—participated in the curriculum. A total of 10 participants completed both the pre- and postsimulation surveys (Table). Of these, six had participated in a simulation before, whereas four had no experience with this learning format. None of the students had any significant prior WM experience.

**Table.**
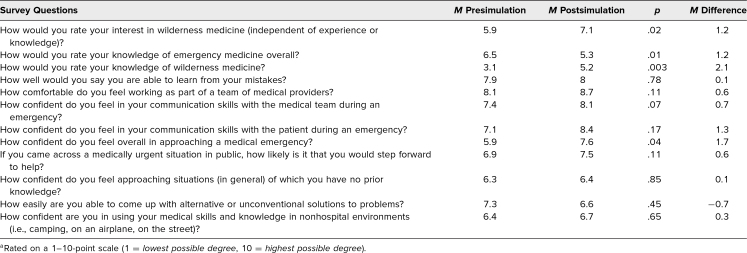
Participant Survey Responses Before and After Wilderness Medicine Simulation (*N* = 10)^a^

The general response was positive. Students indicated that they really enjoyed the day, and 100% of them agreed that the scenarios were helpful in learning to approach wilderness emergency situations. Students found the curriculum to be appropriate for their level of education, specifically ranking the case content a mean 8.9 points. The majority of participants (80%) report feeling more prepared to approach all five case scenarios in the future based on the information and skills they gained. Additionally, 100% of participants answered “yes” when asked if they thought simulation was an effective method of teaching WM. In postsimulation survey comments, one student commented that “the scenarios were very realistic to what we could encounter when hiking… I appreciated being able to take a history that isn't in the context of a clinic.”

The intervention did stimulate some interest in WM. Students' reported interest in WM increased pre- to postsimulation by an average of 1.1 points per individual, from a group mean rating of 5.9 points to 7.1 points (*p* = .02). Furthermore, students' self-reported knowledge of WM increased by a mean 2.1 points, from a group mean rating of 3.1 points to 5.2 points (*p* = .003). This was compared to students' self-reported knowledge of emergency medicine overall, which showed no significant difference after the intervention. As one participant reported, the day was “super informative, especially [for] a novice.”

Following participation in the curriculum, participants' confidence in approaching a WM emergency overall increased by 17%, from a mean rating of 5.9 points to 7.6 points. Additionally, when asked about their confidence level in using their medical skills and knowledge in nonhospital environments, participants reported a postsimulation average score of 6.7 points, which was a slight increase (mean increase 0.3 points) from presimulation.

This curriculum was repeated successfully with a new cohort of student leaders in a similar wilderness setting for an additional 15 medical student participants. This demonstrates the ease of implementation of the curriculum with a new group of instructors and students.

## Discussion

We report the successful implementation of five simulation cases as a tool to teach WM to undergraduate medical students in an urban setting. The curriculum covered topics of anaphylaxis, trimalleolar fracture splinting, traumatic bleeding control, hypothermia, and a choking child. There is a notable lack of pediatric case content for WM education. We hope to add to this catalog with our case and increase interest in expanding this topic. Notably, these cases may function as standalone modules, which increases their adaptability for use by educators who do not wish to implement an hours-long curriculum.

The survey results were largely positive among the student participants who responded: 100% of respondents affirmed that simulation is an effective method of learning WM. Most participants reported feeling more prepared to approach all five case scenarios and ranked the content as appropriate for their level of education. Additionally, participants reported an increase in interest and knowledge of WM. Survey results also indicated a 17% increase in self-reported overall confidence in approaching medical emergencies. However, when asked if they felt more comfortable applying the knowledge they gained in these simulations to similar clinical cases in a hospital setting, only four of the 10 students reported feeling more comfortable approaching all five scenarios. This indicates potentially limited translation of WM skills and knowledge to a nonwilderness (i.e., hospital) setting.

Limitations to this educational intervention include survey size, and the survey's reliance on participant self-evaluation. The event was voluntary with limited spots, inherently decreasing the size of the survey pool. The survey was written by the four authors of this report; it was not previously published, nor did it undergo validity testing. As such, results should function as a narrative review and a subjective self-evaluation, rather than a precise measurement of success.

As this was the first iteration of this curriculum, we learned valuable lessons that will inform future replication efforts. One important consideration is transportation. In cases where participants do not have vehicles to travel to the wilderness site, coordinating group transportation will be a complicator. Another consideration is the provision of food and water for 20–30 individuals who are spending hours outdoors; we provided catered lunch and water.

While this curriculum served as a strong introduction to the field, there is potential for future expansion. Participants' feedback indicated a desire for more complexity in the cases, particularly in relation to wilderness considerations. Elements that would enhance this curriculum include managing environmental challenges such as heavy rain or extreme heat, and safe evacuation of the patient in uneven terrain or austere conditions. Additionally, risk to caregivers is a theme in WM that we did not actively integrate into our curriculum. Future expansions are encouraged to explore factors that complicate rescue efforts, such as injury to the provider, fatigue, or supply management.

In this educational intervention, we established the feasibility of implementing five innovative WM cases designed for the education of medical students. Additionally, we demonstrated the effectiveness of case-based simulation as a compelling teaching method for WM. Feedback emphasized the value of the faculty debrief at the end of each case and highlighted that this was a key factor in the students' learning. This approach not only enhanced student engagement but also significantly improved their confidence and competence in managing medical emergencies in remote settings, underscoring the importance of providing WM education to this population of learners. The results of our novel curriculum pilot support the implementation of simulation-based WM curricula and may prompt broad opportunities for future development.

## Appendices


WM Case 1.docxWM Case 2.docxWM Case 3.docxWM Case 4.docxWM Case 5.docxPre- and Postsurvey.docxPrebriefing and Learner Training Materials.docxCommon Curriculum Clinical Objectives.docx
*All appendices are peer reviewed as integral parts of the Original Publication.*
