# Supplementary material for: Implementation of a Novel Wilderness Medicine Simulation Course for Medical Students
Source: MedEdPORTAL. 2025 Jun 9;21:11526. doi: 10.15766/mep_2374-8265.11526 (PMC12146433; doi:10.15766/mep_2374-8265.11526)
Supplement: Supplementary file 1 — WM Case 1.docxWM Case 2.docxWM Case 3.docxWM Case 4.docxWM Case 5.docxPre- and Postsurvey.docxPrebriefing and Learner Training Materials.docxCommon Curriculum Clinical Objectives.docx [file mep_2374-8265.11526-s001.zip › D. WM Case 4.docx]

This appendix is to be used to guide the flow of each simulated case scenario. The “instructor notes – changes and case branch points” and “ideal scenario flow” sections provide especially detailed instructions on how the simulation actors and facilitators should respond to different actions by the learners. Key learning objectives are listed on the first page.

| **Appendix D: Case 4: Hypothermia**  **SIMULATION CASE TITLE:** *Hypothermia and Concussion: Wilderness Medicine Emergency Simulation for Medical Students*  **AUTHORS:** Sophie Redpath; Katherine Sprengel; Kira Palazzo | |
| --- | --- |
| **PATIENT NAME: Willa Gomez**  **PATIENT AGE: 22**  **CHIEF COMPLAINT: “I’m so cold”** | |
|  | |
| **Brief narrative description of case** | You are with a group hiking up a trail when you come across a lone young woman who is on the ground shivering. She is wearing wet clothes and holding her head. She is laying at the bottom of a small hill, near a stream.  *The anticipated interventions of the responders include: (1) assessing the scene for safety; (2) assessing the patient, recognizing and initiating treatment for hypothermia and concussion; (3) activating the emergency medical system (EMS) and transporting the patient to safety; and (4) utilizing TeamSTEPPS techniques for effective teamwork and communication.*  *Anticipated interventions include ongoing assessment of ABC’s, management of hypothermia and concussion, effective communication, and activation of EMS with safe transport to a higher level of care.*  The patient stabilizes after escalating therapy. |
| **Primary Learning Objectives** | By the end of this activity, learners will be able to:   1. Assess the scene for safety prior to responding to an injured or incapacitated patient. 2. Effectively activate the Emergency Medical System (EMS) in a remote wilderness setting and safely transport and sign out the patient. 3. Demonstrate effective teamwork and communication skills while managing an emergency in a remote setting. |
| **Critical Actions** | ***Crucial:***   1. Assess and constantly reassess the scene for safety 2. Primary, secondary survey 3. Recognize, treat hypothermia (move patient away from water, provide shelter, replace wet clothing with dry) 4. Recognize signs and symptoms, and management of concussion. 5. Activate EMS as soon as cell service is available 6. Assign clear team roles and responsibilities 7. Communicate effectively as a team, including using directed, closed-loop communication 8. Give comprehensive patient sign-out to Medic |
| **Learner Preparation** | Learners will be briefed prior to the exercise regarding the availability of simulated cellular service, the availability of simulated EMS teams, and how to access and activate these systems in the simulated outdoor wilderness environment |

| **Initial Presentation** | |
| --- | --- |
| **Initial vital signs** | HR: 140 RR: 20 Temp: cool to touch |
| **Overall Appearance** | Patient is lying alone next to a stream, in wet clothing, shivering, whimpering and holding her forehead. She is notably upset and has cold, pale fingers. |
| **Actors and roles in the room at case start** | Group of 4 hikers (medical students, residents or fellows) respond to injured patient on trail and divide into roles:  Hiker #1: Team lead  Hiker #2: Survey  Hiker #3: Helper who performs patient interventions (ie: sets up shelter, warms and hydrates patient, concussion assesment, etc.)  Hiker #4: Activates EMS, then helps Hiker #3.  Simulated injured patient: Full body adult-size manikin with ability to change vitals including peripheral pulses, capillary refill. If manikin unavailable or if more practical in a wilderness setting, patient may be enacted by an instructor/helper.  Instructor #1: Simulation instructor who will also act as debriefer.  Instructor #2: If a 2^nd^ instructor is available, cast them as EMS dispatch on the phone and/or EMS provider that arrives to scene. |
| **HPI** | Instructor #1 volunteers vignette:  Patient is a 22-year-old female who was out hiking. She is a young poet from Florida and decided to go on a hike alone for some writing inspiration. She is found alone, cold and injured lying next to a stream. Her teeth are chattering and she is somewhat confused but is able to provide a basic history.  Simulated injured patient: when asked about leading events (SAMPLE):  **S**igns/symptoms - shivering, tearful, localizing pain to right side of forehead  **A**llergies - none  **M**edications - none  **P**ast medical / surgical history - previously healthy, no prior surgeries  **L**ast meal: 3 hours ago (scrambled eggs, fruit and a coffee)  **E**vents leading to incident: Was jumping over a river stream and just missed the bank causing her to fall into the water. In doing so, she hit her head on a rock on the bank, her vision went “fuzzy with twinkly stars” and she does not remember how long she has been sitting there, but knows her hike started at around 8 am. She does not have any other musculoskeletal injuries. Did not bring water, food, medical pack or extra layers. Cell phone fell out into river and died.  Family history - none  If asked for review of systems:  Feels cold, wet, has a severe headache and is sensitive to light and noise. Cannot feel her fingers or toes as they have gone numb. Unsteady on her feet as she is quite dizzy and a little nauseous.  If asked about home environment/social history:  Is staying with a local writers immersion workshop in a dormitory, and did not tell them when she’d return from her hike. She does not recall their phone number by heart but remembers the general neighborhood of her dorm. |
| **Physical Examination** (initial impression) (primary and secondary assessment) | |
| **General** | Wearing wet clothing and hiking boots.  Alert, responsive.  Breathing a little faster than normal.  Is pale and is shivering. |
| **HEENT** | Patent airway, lips are pale and dry, no lip/tongue swelling.  Prominent hematoma to R forehead, no other obvious head trauma or bleeding.  PERRLA (*if have pen light to assess)* |
| **Neck** | Supple. |
| **Lungs** | Respiratory rate 20-25 breaths per minute, no audible abnormal air sounds. |
| **Cardiovascular** | Tachycardic, no murmurs/rubs/gallops. Radial pulses symmetric. |
| **Abdomen** | Soft, non-tender, non-distended.  No obvious trauma to abdomen. |
| **Neurological** | Somewhat confused, but speaking in full sentences.  Sensation, motor, cerebellar, tone, reflexes intact and symmetric.  No vertebral point tenderness. |
| **Skin** | Wet, cold, pale skin. Shivering. Capillary refill 3-4 seconds (slow).  No obvious bleeding.  Scattered superficial abrasions on bilateral hands.  No rash. |
| **Musculoskeletal** | Hiking boots and socks wet, cannot feel her toes. |
| **Psychiatric** | Upset (crying) but cooperative, non-combative. |

| **Instructor Notes - Changes and CASE Branch Points** | | |
| --- | --- | --- |
| **Intervention / Time point** | **Change in Case** | **Additional Information** |
| Hikers come across injured patient. |  | Patient is shivering, sitting down and tries to call out but then grabs her head in pain. |
| Hikers stop and assess for safety, prior to approaching patient to offer help. | Patient is alert but confused | Patient greets hikers slowly, asking “who are you? Why am I so wet?” to convey confusion. She appears to be bothered by looking up into the light and is squinting and shading her eyes. |
| Hikers help move patient away from stream. |  | Patient is confused but cooperative. |
| Hikers divide into roles:  Hiker #1: Team lead  Hiker #2: Survey  Hiker #3: Helper who performs patient interventions  Hiker #4: Activates EMS, then helps Hiker #3 |  |  |
| Hikers complete primary and secondary assessment. | Patient is alert, somewhat irritable and confused but consolable.  Is wet and shivering.  Is breathing a bit fast, with intact and symmetric peripheral pulses.  Skin is pale and cool to touch with delayed capillary refill. | Patient is initially irritated at the physical exam but is calmed when the situation is explained to her. |
| Evaluate that patient has hypothermia by noting her skin is pale, cool to touch, delayed capillary refill, and the patient is shivering.  Team assessment of useful items, ie: shelter, blankets, food, warm/sugary beverages, first aid items, meds.  Ask Patient to assist their efforts to remove her wet clothing and provide dry blankets/jackets/whatever is available. | Patient stops shivering. |  |
| Evaluate that the patient has a concussion, manage symptoms where possible (i.e. giving her sunglasses to wear). | Patient is holding her head and seems sensitive to loud noises and direct light. | She repeats “my head really hurts” several times, but denies any neck pain or other injuries. She is a bit delayed in her responses but is able to respond to all questions. |
| If a thermos is available in the participant’s packed daypack or in the backpack provided by the sim instructor, provide patient with warm, sugar-sweetened beverages to drink. | As patient warms up and can feel her toes better she is able to stand without help. If asked she states that she still feels “out of it” or has “brain fog.” | Wlila continues holding her head localized to the hematoma on the right side. but seems more comfortable now that she is warm, hydrated, and dry. |
| Create an evacuation plan and call for EMS when possible if not already done. |  |  |
| EMS greet group at trailhead.  Hikers give sign-out of pertinent information. |  | Patient greets EMS, thanks the hikers, and is happy to get into the warm ambulance. |

**Ideal Scenario Flow**

- The hikers are hiking down the trail and come across the patient on the trail. They assess the scene for safety before approaching the patient.
- The hikers are able to calm the young woman by assuring her that they are there to help and continuing to communicate clearly and empathetically with the patient.
- They divide into team roles (team leader, survey, caller for help, and provider of patient care).
- They perform an initial patient assessment and continuously reassess along the way. They acknowledge that she is at risk for hypothermia and has a painful and evident hematoma to the right forehead.
- They move her away from the stream and proceed to gather and utilize whatever gear they brought in their daypack or is otherwise available in a pack provided by the facilitator.
- They provide a shelter, assist her in removing her own wet clothing and boots, and provide her with dry clothing/blankets from their own supply. They give her warm, sugary beverages to drink, if available.
- They complete a full body exam and a neurological exam to assess the extent of the concussion symptoms. Acknowledge that the patient has a hematoma, appears somewhat confused, is wearing wet clothes and shivering. A full assessment is indicated, ie: assess all clothing for wetness/temperature, examine her neck/back for injury, perform a neuro exam suspecting concussion.
- They support the patient's evacuation by supporting her as she walks to the trailhead, or carrying in a litter if they determine she is too altered to self evacuate (depending on the training of the participants).
- They activate EMS by pretending to call 911 on their cell phones once they get service (the facilitator will tell them when they are in service range and remind them not to actually call 911) and the scenario ends when EMS arrives and the participants give a thorough patient sign-out.

**Anticipated Management Mistakes**

- Failure to obtain pertinent history and physical and recognize the patient’s condition. If the learner does not obtain the salient points of the history and physical that suggest hypothermia and head injury, then the simulated patient actor can volunteer this information to the learner by exaggerating the symptoms. Likewise, the hike leader/facilitator can make a suggestion (ie: “she looks like he’s shivering, where is her water bottle, why is she squinting at the light and confused”)
- Failure address hypothermia: Once determined that it is safe to approach the patient and that the patient is alert and cooperative, the team must recognize the risk of hypothermia and help her move away from the water before continuing the assessment and management of the learning objectives. If the learners do not make the effort to remove the cold clothing, provide warm clothing and shelter, and give warm sugary fluids, then the patient can become more altered and somnolent. The hike leader/facilitator can intervene with a prompt (ie: “here is my backpack full of warm clothes and fluids.”)
- Failure to recognize and assess concussion: Once the hematoma is identified and the mechanism of injury identified, discussion of concussion symptoms and management should proceed. There are several concussion assessment tools and protocols that are acceptable for this evaluation. Consider using NEXUS.
- Discussion of C-collar administration: Once students have identified and evaluated concussion/head trauma and mechanism of injury, there may be a conversation about safe evacuation and the use of a C-collar. The patient denies neck pain and has no vertebral tenderness, therefore in this case it is not indicated as a necessary intervention.
- Failure to call for help. If this occurs, the facilitator eventually can provide the cell phone or indicate that cell service is working.
- Failure to extract patient. This is a step that will change, depending on the context. If the hikers decide the patient is too altered to self evacuate, they can create a litter (if they have the experience), if the litter is inadequate or unstable the patient may insist on walking out herself, supported by the hikers.
- Discussion Points: Important elements to consider when managing hypothermia in the wilderness including creating a barrier between the patient and ground, active rewarming techniques, nutritional support, and recognition of signs of decompensation.
