# Supplementary material for: Implementation of a Novel Wilderness Medicine Simulation Course for Medical Students
Source: MedEdPORTAL. 2025 Jun 9;21:11526. doi: 10.15766/mep_2374-8265.11526 (PMC12146433; doi:10.15766/mep_2374-8265.11526)
Supplement: Supplementary file 1 — WM Case 1.docxWM Case 2.docxWM Case 3.docxWM Case 4.docxWM Case 5.docxPre- and Postsurvey.docxPrebriefing and Learner Training Materials.docxCommon Curriculum Clinical Objectives.docx [file mep_2374-8265.11526-s001.zip › E. WM Case 5.docx]

This appendix is to be used to guide the flow of each simulated case scenario. The “instructor notes – changes and case branch points” and “ideal scenario flow” sections provide especially detailed instructions on how the simulation actors and facilitators should respond to different actions by the learners. Key learning objectives are listed on the first page.

| **Appendix E: Case 5: Choking Child**  **SIMULATION CASE TITLE:** *Infant Choking Scenario: Wilderness Medicine Emergency Simulation for Medical Students*  **AUTHORS:** Katherine Sprengel; Sophia Redpath; Kira Palazzo | |
| --- | --- |
| **PATIENT NAME:** Manny  **PATIENT AGE:** 1y  **CHIEF COMPLAINT: “My child isn’t answering me!”** | |
|  | |
| **Brief narrative description of case** | You come across a parent hiking with their 1yo child in a hiking backpack. As you approach, the parent is trying unsuccessfully to talk with the child. They call out to you – “Can you see my son? Is he ok? He’s not answering me!”  The anticipated interventions include: 1) assessing the scene for safety, 2) assess child on back to determine safety for removal, 3) remove child and assess ABCs, 4) recognize choking, perform back blows and mouth sweeps as necessary, 5) effective management of a pediatric patient vomiting (ie: airway management) 6) utilize effective teamwork and communication with the team and with the parent.  The patient vomits and stabilizes after 3 rounds of back blows therapy. |
| **Primary Learning Objectives** | By the end of this activity, learners will be able to:   1. Assess the scene for safety prior to responding to an injured or incapacitated patient. 2. Assess the patient, recognize a choking child and provide effective management. 3. Effectively activate the Emergency Medical System (EMS) in a remote wilderness setting and safely transport. 4. Demonstrate effective teamwork and communication skills while managing an emergency in a remote setting. |
| **Critical Actions** | ***Crucial:***   1. Assess and constantly reassess the scene for safety 2. Primary, secondary survey 3. Safe removal of child from backpack 4. Recognize, treat choking (5 back blows followed by 5 chest thrusts until object is expelled from mouth) 5. effective management of child post - vomiting 6. Activate EMS as soon as cell service is available 7. Assign clear team roles and responsibilities 8. Communicate effectively as a team, including using directed, closed-loop communication 9. Give comprehensive patient sign-out to Medic |
| **Learner Preparation** | Overview of how to help a child who is choking given during pre-briefing. |

| **Initial Presentation** | |
| --- | --- |
| **Initial vital signs** | HR: 150 RR: unable to measure Temp: normal |
| **Overall Appearance** | Patient appears distressed. He is not making any sound, though he is attempting to cough. His lips are turning slightly blue. |
| **Actors and roles in the room at case start** | Group of 4 hikers (medical students) respond to injured patient on trail and divide into roles:  Hiker #1: Team lead  Hiker #2: Survey and parent communication  Hiker #3: Helper who performs patient interventions (ie: assists with extraction from backpack, backblows, mouth sweeps, patient positioning)  Hiker #4: Activates EMS, then helps Hiker #3.  Simulated injured patient: Infant-size high-fidelity Manikin in a hiking backpack.  Simulated parent: Simulation instructor will act as the distraught parent.  Instructor #1: Simulation instructor who will act as the distraught parent, and also act as debriefer.  Instructor #2: If a 2^nd^ instructor is available, cast them as EMS dispatch on the phone. If there is only one instructor available, they will play both roles. |
| **HPI** | Patient is a 1 year old boy on a hike with his parent, being carried in a backpack. Patient recently started eating solid foods, and his mom has been feeding him snacks over her shoulder. When you arrive she is holding a bag of grapes and trying to talk to Manny over her shoulder. He is making no noise but appears to be in moderate distress and is attempting to cough.  Simulated injured patient: when asked about leading events (SAMPLE):  **S**igns/symptoms - not making any noise, lips are blue, he appears visibly frightened  **A**llergies - none  **M**edications - none  **P**ast medical / surgical history - G1P1 vaginal birth no complications, no prior surgeries. He had a cold a few weeks ago that the parents treated at home with supportive care.  **L**ast meal: 2 hours ago (mushed up banana with peanut butter and soft bread)  **E**vents leading to incident: Mom and patient go on day hikes together often, today was no different. He has been more hungry recently so she has been trying out new foods with him while at home, and today was feeding him small bites of her snacks over her shoulder while hiking.  Family history - his older sister has asthma, for which she uses an inhaler.  If asked for review of systems:  Manny is visibly upset, shaking his arms and head, and coughing silently but unsuccessfully. Last urinated two hours ago before start of hike. No obvious trauma or deformities, no bleeding.  If asked about home environment/social history:  Manny lives in a home 20 minutes away with his parent (present) and their spouse. |
| **Physical Examination** (initial impression) (primary and secondary assessment) | |
| **General** | Wearing athletic clothing, in his parent’s carrying backpack.  Responsive, but in distress and unable to make sound.  Not breathing. |
| **HEENT** | Not breathing. Lips are turning blue.  No apparent trauma to head or neck.  Eyes: PERRLA (if have pen light to assess) |
| **Neck** | Supple. No masses noted. |
| **Lungs** | No respiratory rate, patient is unable to breathe. Wheezing heard on auscultation. |
| **Cardiovascular** | Tachycardic, no murmurs/rubs/gallops. Radial pulses symmetric. |
| **Abdomen** | Soft, non-tender, non-distended.  No obvious trauma to abdomen. |
| **Neurological** | Alert, unable to speak or make noise.  Sensation, motor, cerebellar, tone, reflexes intact and symmetric. |
| **Skin** | Dry, warm. Capillary refill 1 second.  No obvious bleeding.  Lips are slightly blue.  No rash. |
| **Musculoskeletal** | No obvious trauma or injury. No wincing or guarding when you remove him from the backpack. |
| **Psychiatric** | Visibly panicking. |

| **Instructor Notes - Changes and CASE Branch Points** | | |
| --- | --- | --- |
| **Intervention / Time point** | **Change in Case** | **Additional Information** |
| Hikers come across parent and patient. | Patient has vitals noted above | The parent asks the hikers if her baby looks okay, but she is not yet aware of the gravity of the situation. |
| Hikers stop and assess for safety, prior to approaching patient to offer help. Hikers begin to assess baby while in backpack. | Patient waves his arms around and appears to be clawing at his throat, is not able to make any noise. | The parent tries to speak to her child but becomes more distressed as her baby is still is not responding. |
| Hikers divide into roles:  Hiker #1: Team lead  Hiker #2: Survey  Hiker #3: Helper who performs patient interventions  Hiker #4: Activates EMS, then helps Hiker #3 |  |  |
| Hikers remove patient from his backpack. |  | The parent is becoming increasingly distraught when hikers remove patient and she sees him choking. She tries to grab patient from them but backs off when told by hikers they are here to help. |
| Evaluate that patient is choking by noting his lips are blue, he is not breathing, and is attempting to cough unsuccessfully. The team notes the bag of grapes in the parent’s hand. | Instructor informs them that patient’s lips are blue, and he does not appear to be breathing. | The parent begins crying out about her baby’s blue lips and begs the hikers to help him. |
| Turn child over with head towards the ground but supported, deliver back blows in a down and out motion. (x5)  Begin chest thrusts: turn baby face-up, place two fingers on breastbone, and deliver thrusts. (x5) |  | If prompted about the grapes, the parent responds “Oh no, I was eating my grapes and he asked for one so I handed one back. I wasn’t thinking. Oh my god, is my baby going to be okay?” |
| Following 3 rounds of chest thrusts and back blows, patient vomits and begins coughing forcefully. |  |  |
| Hikers complete PALS initial assessment, primary and secondary assessment. |  |  |
| Hikers contact EMS via phone and provide pertinent information over phone. Direct parent to the trailhead to meet them, or accompany her. |  | The parent says, “Thank you so much for saving my son!” |

**Ideal Scenario Flow**

- Hikers proceed down the trail and encounter a woman carrying a child in a backpack.
- They assess the scene for safety before approaching the patient.
- The parent is trying to speak to the child (manikin) in her backpack but he is not answering her and is increasingly distressed
- They approach the parent and ask if they can help, as her baby appears to be in distress. They divide up team roles (leader, survey, call for help, patient care) and perform an initial assessment.
- Hikers note that the baby is not breathing and acknowledge that the risk is choking. They remove the baby from the backpack and reassess.
- Begin administering chest thrusts and back blows. After 3 rounds, the baby vomits and begins coughing. They maintain his airway and reassess his breathing.
- Pretend to activate EMS by calling 911 on their cell phones, and give a sign-out of pertinent information over the phone.
- Perform appropriate handoff to ‘EMS personnel’ once they arrive

**Anticipated Management Mistakes**

- Failure to obtain pertinent history and physical and recognize the patient’s condition. If the learner does not obtain the salient points of the history and physical that suggest choking, the parent can prompt with , “Do his lips look blue to you?”. The instructor can also offer suggestions like, “Have you checked the airway?” Finally, the parent can show her grapes and say, “I just gave him one of these to eat, do you think that caused this?”
- Failure address choking: Once the team recognizes that the child is choking, they must assess for injury and establish that is it is safe to remove him from the backpack to continue the assessment and management of the learning objectives. If the learners do not make the effort to remove Manny from the backpack, his panicking and weak coughing can become worse. The instructor can prompt (“It may be easier to assess the patient more fully if you remove him from the backpack.”)
- Failure to correctly respond to a pediatric choking scenario: Common mistake is to reach into child’s mouth to try to remove object. If this is done, it can push the foreign object further into the throat. Another common mistake is to try to do the Heimlich maneuver on the child. Back blows followed by chest thrusts is the proper procedure.
- Failure to call for help. The parent can prompt with, “Should I take him somewhere else? Do you have a phone to call for help?”
