# Supplementary material for: Implementation of a Novel Wilderness Medicine Simulation Course for Medical Students
Source: MedEdPORTAL. 2025 Jun 9;21:11526. doi: 10.15766/mep_2374-8265.11526 (PMC12146433; doi:10.15766/mep_2374-8265.11526)
Supplement: Supplementary file 1 — WM Case 1.docxWM Case 2.docxWM Case 3.docxWM Case 4.docxWM Case 5.docxPre- and Postsurvey.docxPrebriefing and Learner Training Materials.docxCommon Curriculum Clinical Objectives.docx [file mep_2374-8265.11526-s001.zip › F. Pre- and Postsurvey.docx]

**APPENDIX F: Pre- and Postsurvey**

*Instructions for use: These survey questions are an optional component of this simulation curriculum. They can be administered to participants before and after the simulation day to subjectively assess participant interest and learning.*

**Pre-Simulation Survey**

1. What are the last 4 digits of your phone numbers (to be used as study ID)? (text box)
2. Graduation year? (drop-down: 2026, 2025, 2024)
3. Have you participated in a simulation (SIM) event before (in the emergency room or otherwise)? (Y/N)
4. Do you have previous experience in wilderness medicine? (Y/N)
   1. If so, what? (text box)

**Repeat Pre-Simulation and Post-Simulation Survey Questions**

In all of the following questions, please rate on a scale from 1-10 (10 represents the highest possible degree and 1 represents the lowest).

1. How would you rate your interest in wilderness medicine (independent of experience/knowledge)?
2. How would you rate your knowledge of emergency medicine?
3. How would you rate your knowledge of wilderness medicine?
4. How well would you say you are able to learn from your mistakes?
5. How comfortable do you feel working as part of a *team* of medical providers?
6. How confident do you feel in your communication skills during a medical emergency?
   1. With your team?
   2. With the patient/family?
7. How confident do you feel overall in approaching a medical emergency?
8. If you came across a medically urgent situation in public, how likely is it that you would step forward to help?
9. How confident do you feel approaching situations of which you have no prior knowledge?
10. How easily are you able to come up with alternative or unconventional solutions to problems?
11. How confident are you in using your medical skills/knowledge in non-hospital environments (i.e. camping, on an airplane, on the street)?

**Post-Simulation Survey**

1. What are the last 4 digits of your phone number (to be used as study ID)? (text box)
2. Did you enjoy today’s simulation? (Y/N)
3. Any recommendations for improvement? (Free text)
4. Please rate the curriculum presented in the cases today from 1-10
5. Please rate the overall simulation day event from 1-10
6. Do you feel that today’s activities were helpful to you in learning to approach wilderness emergency situations? (Y/N)
   1. Did the actors and props help simulate real cases? (Y/N)
7. Do you think SIM is an effective way to learn medicine? (Y/N)
   1. Specifically wilderness medicine? (Y/N)
8. Cases
   1. Do you feel more prepared to approach the following cases, as compared to before the SIM? (All Y/N)
      1. Hypothermia
      2. Anaphylaxis
      3. Fracture
      4. Arterial bleed
      5. Choking child
   2. Do you feel more prepared to do so in a non-hospital environment? (Y/N)
   3. Do you feel more comfortable in management of the situational considerations of wilderness medical cases (ie: scene safety, environmental safety/exposure and maintenance of safety, evacuation techniques and considerations, considerations of extended time frames) (Y/N)
