# Supplementary material for: Implementation of a Novel Wilderness Medicine Simulation Course for Medical Students
Source: MedEdPORTAL. 2025 Jun 9;21:11526. doi: 10.15766/mep_2374-8265.11526 (PMC12146433; doi:10.15766/mep_2374-8265.11526)
Supplement: Supplementary file 1 — WM Case 1.docxWM Case 2.docxWM Case 3.docxWM Case 4.docxWM Case 5.docxPre- and Postsurvey.docxPrebriefing and Learner Training Materials.docxCommon Curriculum Clinical Objectives.docx [file mep_2374-8265.11526-s001.zip › G. Prebreifing and Learner Training Materials.docx]

**APPENDIX G: Pre-briefing and Learner Training Materials**

*Instructions: These materials are recommended to be sent to participants in the days prior to the simulation so they can prepare. This is an optional component of the curriculum and should be used at the discretion of the facilitators depending on the education level of learners.*

The materials sent to learners before the event included suggested personal items to bring, and three optional instructional videos demonstrating techniques for wilderness patient evacuation (1), and using SAM splints and triangle bandages to create leg splits (2) and shoulder splints (3).

Participants were also pre-briefed before beginning the simulation exercise. Pre-briefing topics included how to use the included items (a full list can be found under Equipment heading), the availability of simulated cellular service, the availability of simulated EMS teams, and how to access and activate these systems in the simulated outdoor wilderness environment.

Debriefing sessions were held immediately after completion of each case, and these were led by Emergency Medicine physicians experienced in simulation based medical education. Given the limited resources available in the outdoor setting, it was decided to use a verbal debrief in place of formal presentation following each scenario. Debriefing faculty utilized typical debriefing formats and were provided with key debriefing points to share with learners (case-specific critical actions and anticipated management mistakes are embedded in each case).

VIDEO REFERENCES:

1. “Search and Rescue: Carrying an Injured Patient” *YouTube,* Uploaded by MedWild - Wilderness Medicine, Survival, Rescue, June 29 2015 <<https://www.youtube.com/watch?v=sMeqn3P3WGc>>
2. “Wilderness Medicine | Leg Splints” *YouTube,* Uploaded by NOLS Wilderness Medicine August 7 2012 <<https://www.youtube.com/watch?v=TacM-3A5Ozk>>
3. “Wilderness Medicine: Shoulder Splinting” *YouTube,* Uploaded by MedWild - Wilderness Medicine, Survival, Rescue, December 23 2012 <<https://www.youtube.com/watch?v=gtdNhIEqhvQ>
