# Supplementary material for: Implementation of a Novel Wilderness Medicine Simulation Course for Medical Students
Source: MedEdPORTAL. 2025 Jun 9;21:11526. doi: 10.15766/mep_2374-8265.11526 (PMC12146433; doi:10.15766/mep_2374-8265.11526)
Supplement: Supplementary file 1 — WM Case 1.docxWM Case 2.docxWM Case 3.docxWM Case 4.docxWM Case 5.docxPre- and Postsurvey.docxPrebriefing and Learner Training Materials.docxCommon Curriculum Clinical Objectives.docx [file mep_2374-8265.11526-s001.zip › H. Common Curriculum Clinical Objectives.docx]

**APPENDIX H: Common Curriculum Clinical Objectives**

*Instructions: This appendix provides an overview of the key curriculum objectives common to each of the simulated cases, and for the entire curriculum. It can be used at the discretion of the facilitators in assessing participant learning.*

| Objective | Critical Action |
| --- | --- |
| Clinical Objective #1  Safely approach a medical emergency in a wilderness setting | - Assess scene for safety (i.e. environmental hazards, weather, animals, human factors) - Approach patient in non-threatening way using appropriate communication |
| Clinical Objective #2  Communicate effectively within the team and with the patient/family | - Utilize effective communication within the provider team - Keep patient updated with medical management in a level of detail appropriate to their level of understanding - Obtain consent to treat and proceed when possible |
| Clinical Objective #3  Provide medical management as appropriate for each individual case | Specific medical management of each case is outlined in detail in APPENDICES A-E |
| Clinical Objective #4  Discuss safe evacuation technique and perform sign-out to EMS | - Assess both the scene and the status of the patient to determine the safest manner of evacuating - Assess and execute the best manner of EMS activation |
