# Supplementary material for: Implementation of a Novel Wilderness Medicine Simulation Course for Medical Students
Source: MedEdPORTAL. 2025 Jun 9;21:11526. doi: 10.15766/mep_2374-8265.11526 (PMC12146433; doi:10.15766/mep_2374-8265.11526)
Supplement: Supplementary file 1 — WM Case 1.docxWM Case 2.docxWM Case 3.docxWM Case 4.docxWM Case 5.docxPre- and Postsurvey.docxPrebriefing and Learner Training Materials.docxCommon Curriculum Clinical Objectives.docx [file mep_2374-8265.11526-s001.zip › A. WM Case 1.docx]

This appendix is to be used to guide the flow of each simulated case scenario. The “instructor notes – changes and case branch points” and “ideal scenario flow” sections provide especially detailed instructions on how the simulation actors and facilitators should respond to different actions by the learners. Key learning objectives are listed on the first page.

| **Appendix A: Case 1: Anaphylaxis**  **SIMULATION CASE TITLE:** *Anaphylaxis with Epi-pen use: Wilderness Medicine Emergency Simulation for Medical Students*  **AUTHORS:** Sophia Redpath; Katherine Sprengel; Kira Palazzo | |
| --- | --- |
| **PATIENT NAME: Tom Cunningham**  **PATIENT AGE: 22**  **CHIEF COMPLAINT: “I think I accidentally ate a peanut!”** | |
|  | |
| **Brief narrative description of case** | You are with a group hiking on a trail when you come across a young man alone who is sitting down and appears to be in mild distress. He is sitting on the side of the trail using both arms for support and leaning forward (tripod-ing). He notices you and waves you over to try to ask for help between labored breaths.  The anticipated interventions of the responders include: (1) assessing the scene for safety; (2) assessing the patient, recognizing and initiating treatment for a severe allergic reaction including using the epi-pens accurately; (3) transporting the patient to safety and if applicable calling EMS.  Anticipated interventions include ongoing assessment of ABCs, ongoing management of allergic reaction, effective communication, and activation of EMS with safe transport to a higher level of care.  The patient stabilizes after 2 epi-pen injections and is able to walk on his own. |
| **Primary Learning Objectives** | By the end of this activity, learners will be able to:   1. Assess the scene for safety prior to responding to an injured or incapacitated patient. 2. Assess the patient according to standard emergency response protocols. 3. Quickly recognize a serious allergic reaction; assess and stabilize the patient. 4. Effectively activate the Emergency Medical System (EMS) in a remote wilderness setting and safely transport and sign out the patient. 5. Demonstrate effective teamwork and communication skills while managing an emergency in a remote setting. |
| **Critical Actions** | ***Crucial:***   1. Assess and constantly reassess the scene for safety 2. Primary, secondary survey 3. Recognize, treat anaphylaxis (utilize epi-pen, monitor and maintain patent airway, stabilize for removal with benadryl) 4. Activate EMS as soon as cell service is available 5. Assign clear team roles and responsibilities 6. Communicate effectively as a team, including using directed, closed-loop communication 7. Give comprehensive patient sign-out to Medic |
| **Learner Preparation** | Learners will be briefed prior to the exercise regarding: the availability of simulated cellular service, the availability of simulated EMS teams, and how to access and activate these systems in the simulated outdoor wilderness environment |

| **Initial Presentation** | |
| --- | --- |
| **Initial vital signs** | HR: 120, weak. RR: 20, b/l wheezes |
| **Overall Appearance** | Pt is sitting alone next to the trail in tripod position clearly struggling to breathe. His skin is flushed and his cap refill if checked is slow (hypotensive) |
| **Actors and roles in the room at case start** | Group of 4 hikers (medical students) respond to injured patient on trail and divide into roles:  Hiker #1: Team lead  Hiker #2: Survey and scene safety  Hiker #3: Helper who performs patient interventions (ie: epi pen use, benadryl administration, assists in evacuation)  Hiker #4: Activates EMS, then helps Hiker #3  Simulated injured patient: Full body manikin with capillary refill, pulse, respiratory rate ability; If manikin unavailable or if more practical in a wilderness setting, patient may be enacted by an instructor/helper.  Simulation Facilitator: Additional simulation team member to guide simulation intervention use and determine resource availability |
| **HPI** | Instructor #1 volunteers vignette:  Patient is a 22-year-old male who was out hiking. He is a college student from Texas, here for bachelors degree, and decided to go on a hike alone on his day off. He is found alone, in respiratory distress. He is able to give a partial history between breaths before losing the ability to speak after becoming dizzy and unable to breathe.  Simulated injured patient: when asked about leading events (SAMPLE):  **S**igns/symptoms - tripod position, wheezing, labored breathing  **A**llergies - peanuts. Was told about this allergy as a child, carries an epi pen but has never used it  **M**edications - albuterol as needed  **P**ast medical / surgical history - previously healthy, no prior surgeries  **L**ast meal: 6 hours ago (turkey sandwich), had a granola bar on the trail  **E**vents leading to the incident: Was out on a solo hike and stopped for water and a snack. Reported eating a new brand of granola bar that his roommate gave him. Started walking again but had to stop within a minute due to difficulty breathing. He has an Epi pen but has never used it. He does not have a medical pack or cell service.  Family history - father has asthma  If asked for review of systems:  Skin is flushed, warm. Patient is nauseous and has mild stomach cramps but has not vomited. Urinated before the hike, no current urge to urinate. MSK exam all normal.  If asked about home environment/social history:  Is living in the dorms with one roommate. He told his roommate that he would be home later but did not specify a time or give a specific location of his hike. He does not recall his roommate's phone number by heart. |
| **Physical Examination** (initial impression) (primary and secondary assessment) | |
| **General** | Wearing hiking clothing and boots.  Alert, responsive.  Breathing slowly, wheezing, appears labored.  Is flushed and warm. |
| **HEENT** | Patent airway, mild lip swelling.  No obvious trauma to head or extremities.  PERRLA (*if have pen light to assess)* |
| **Neck** | Supple. |
| **Lungs** | Respiratory rate 13-15 breaths per minute, b/l wheezing |
| **Cardiovascular** | Tachycardic, no murmurs/rubs/gallops. Radial pulses symmetric and weak. |
| **Abdomen** | Soft, non-tender, non-distended.  No obvious trauma to abdomen. |
| **Neurological** | Alert, speaking in full sentences.  Sensation, motor, cerebellar, tone, reflexes intact and symmetric.  No vertebral point tenderness. |
| **Skin** | Dry, flushed, warm. Capillary refill 3-4 seconds.  No obvious bleeding, deformities, or trauma. |
| **Musculoskeletal** | Normal. |
| **Psychiatric** | Clear emotional distress leading into panic. |

| **Instructor Notes - Changes and CASE Branch Points** | | |
| --- | --- | --- |
| **Intervention / Time point** | **Change in Case** | **Additional Information** |
| Hikers come across patient, seated next to a tree. |  | Patient spots hikers and waves hikers over, trying to ask for help between breaths. |
| Hikers stop and assess for safety, prior to approaching patients to offer help. |  | Patient is somewhat calmed by their presence, but continues labored breathing. |
| Hikers divide into roles:  Hiker #1: Team lead  Hiker #2: Survey  Hiker #3: Helper who performs patient interventions  Hiker #4: hikes to service to activate EMS, then helps Hiker #3 |  | Patient asks, gasping for breath, “Can’t breathe…help….” |
| Hikers complete initial assessment, primary and secondary assessment. | Patient is becoming increasingly dizzy and short of breath. Cannot speak more than one word between breaths and there is now visible lip and tongue swelling.  Instructor can point out swelling and physical exam signs. | Patient is able to point to his backpack (where there is an epi pen) but is unable to speak. |
| Hikers find an epi pen in patient’s backpack.  Hikers administer the first Epi-pen dose and continually assess vitals. | Patient improves somewhat, airway is patent and vitals improve somewhat (HR 100, RR 18, no wheezing). Patient is agitated and diaphoretic. | Patient thanks the hikers for their help, now that he is beginning to speak. He is scared and panicky. |
| Evaluate patients vitals and airway, determine if another dose of epi is needed. | Patient starts getting short of breath again, hives return, lips and tongue are markedly swollen again. | Patient gasps “it's coming back,” starts wheezing and becoming more short of breath. |
| The team administers a second dose of epinephrine from their pack (or Patient’s pack), discuss evacuation options should patient improve or deteriorate. | Patient improves with the second dose, airway returns and vitals improve (HR 95, RR 16). | Patient appears more calm, states that he is feeling better and can catch his breath. |
| Team prepares and executes evacuation plan | If prompted, patient is able to swallow and can take a benadryl and walk out |  |
| EMS greet group at trailhead.  Hikers give sign-out of pertinent information. |  | Patient shows immediate relief at the sight of EMS, thanks hikers and is put on O2 on the way to the hospital. |

**Ideal Scenario Flow**

- The simulation starts and the participants set out on a hike then promptly come across an injured hiker in distress. They STOP and assess the scene for safety prior to approaching the injured hiker, specifically noting a lack of environmental or human dangerous situations.
- They are able to calm the young man by assuring him that they are there to help.
- They assign team roles (including team leader, survey, caller for help, and provider of patient care activities).
- They perform an initial patient assessment and continuously reassess along the way. They obtain vitals, history, and perform initial assessment of the patient.
- They note that the patient is in acute respiratory distress, with hives and airway swelling. May also make note of a nut-based granola bar wrapper on the ground. Acknowledge that the patient is having an anaphylactic reaction, begin interventions. They administer the epi pen.
- Recognize increasing HR, respiratory distress and returning hives and oral swelling. They administer a second dose of the epi pen.
- They discuss evacuation options, and after ensuring that the patient is able to swallow, administer benadryl and proceed with a simple accompanied evacuation.
- They pretend to activate EMS by calling 911 on their cell phones once they get service (the facilitator will tell them when they are in service range and remind them not to actually call 911) and the scenario ends when EMS arrives and the participants give a thorough patient sign-out.

**Anticipated Management Mistakes**

- Failure to obtain pertinent history and physical and recognize the patient’s condition. If the learner does not obtain the salient points of the history and physical that suggest allergic reaction then the simulated patient actor can volunteer this information to the learner by exaggerating the symptoms or bringing up the unknown ingredients in the granola bar again. Likewise, the hike leader/facilitator can make a suggestion.
- Failure to provide benadryl as a bridge-therapy: Once it is determined that the patient can walk and swallow a bridge therapy antihistamine should be administered while getting the patient to EMS. If the team fails to provide this, the facilitator may make a suggestion.
- Failure to call for help. If this occurs, the facilitator eventually can provide the cell phone or indicate that cell service is working by getting phone alerts.
- Failure to extract patient. This is a step that will change, depending on the context. If part of a wilderness medicine course, the learners can make a rope litter or use a wheelbarrow carry. If they don’t have those skills, upon splinting the ankle the patient will be able to limp out with support. The simulated patient will be alerted prior to the simulation as to which scenario to expect. If the learners try to carry out the patient without knowing how to, the simulated patient can insist on trying to walk using a cane or an arm as support.
