# Supplementary material for: Implementation of a Novel Wilderness Medicine Simulation Course for Medical Students
Source: MedEdPORTAL. 2025 Jun 9;21:11526. doi: 10.15766/mep_2374-8265.11526 (PMC12146433; doi:10.15766/mep_2374-8265.11526)
Supplement: Supplementary file 1 — WM Case 1.docxWM Case 2.docxWM Case 3.docxWM Case 4.docxWM Case 5.docxPre- and Postsurvey.docxPrebriefing and Learner Training Materials.docxCommon Curriculum Clinical Objectives.docx [file mep_2374-8265.11526-s001.zip › B. WM Case 2.docx]

This appendix is to be used to guide the flow of each simulated case scenario. The “instructor notes – changes and case branch points” and “ideal scenario flow” sections provide especially detailed instructions on how the simulation actors and facilitators should respond to different actions by the learners. Key learning objectives are listed on the first page.

| **Appendix B: Case 2: Trimalleolar Fracture**  **SIMULATION CASE TITLE:** *Trimalleolar fracture with splinting: Wilderness Medicine Emergency Simulation for Medical Students*  **AUTHORS:** Kira Palazzo, Sophia Redpath, Katherine Sprengel | |
| --- | --- |
| **PATIENT NAME:** Chris Topher  **PATIENT AGE:** 30 years old  **CHIEF COMPLAINT: “My ankle hurts so badly and I can’t walk!”** | |
|  | |
| **Brief narrative description of case** | You are with a group hiking up a trail when you come across a lone man who appears to be in pain. He is lying down next to the trail and is holding his right ankle and he is alert. He calls out to you to help him.  The anticipated interventions of the responders include: (1) assessing the scene for safety; (2) assessing the patient, recognizing and initiating treatment for ankle injury; (3) activating the emergency medical system (EMS) and transporting the patient to safety (if possible), and (4) using effective interpersonal communication.  Anticipated interventions include ongoing assessment of ABCs, field stabilization of musculoskeletal injury, effective communication, and activation of EMS with safe transport to a higher level of care.  The patient stabilizes after escalating therapy. |
| **Primary Learning Objectives** | By the end of this activity, learners will be able to:   1. Assess the scene for safety prior to responding to an injured or incapacitated patient. 2. Assess the patient, recognize an injured hiker with an unstable ankle injury and provide effective initial stabilization and management. 3. Effectively activate the Emergency Medical System (EMS) in a remote wilderness setting and safely transport and sign out the patient. 4. Demonstrate effective teamwork and communication skills while managing an emergency in a remote setting. |
| **Critical Actions** | ***Crucial:***   1. Assess and constantly reassess the scene for safety 2. Primary and secondary survey 3. Recognize, and stabilize ankle injury (evaluate for good pulses/sensation, identify no need to reduce, provide adequate splint, assist in carry to safety if patient is unable to bear weight) 4. Activate EMS as soon as cell service is available 5. Assign clear team roles and responsibilities 6. Communicate effectively as a team, including using directed, closed-loop communication 7. Give comprehensive patient sign-out to EMS |
| **Learner Preparation** | Learners will be briefed prior to the exercise regarding the availability of simulated cellular service, the availability of simulated EMS teams, and how to access and activate these systems in the simulated outdoor wilderness environment |

| **Initial Presentation** | |
| --- | --- |
| **Initial vital signs** | HR: 90 RR: 20 Temp: 98.7 |
| **Overall Appearance** | Patient is lying alone next to the trail, groaning and holding his right ankle. He is notably upset. |
| **Actors and roles in the room at case start** | Group of 4 hikers (medical students) respond to injured patient on trail and divide into roles:  Hiker #1: Team lead  Hiker #2: Survey  Hiker #3: Helper who performs patient interventions (ie: sets up shelter, splints ankle, etc.)  Hiker #4: Activates EMS, then helps Hiker #3.  Simulated injured patient: Full body or lower-extremity manikin with palpable lower extremity pulses.; If manikin unavailable or if more practical in a wilderness setting, patient may be enacted by an instructor/helper.  Instructor #1: Simulation instructor who will also act as debriefer.  Instructor #2: If a 2^nd^ instructor is available, cast them as EMS dispatch on the phone and/or EMS provider that arrives to scene. |
| **HPI** | Instructor #1 volunteers vignette:  Patient is a 30-year-old male who was hiking. He is a journalist, and decided to go on a hike alone on his day off. He is found alone and injured lying next to the trail. He is in pain, but can give a detailed history.  Simulated injured patient: when asked about leading events (SAMPLE):  **S**igns/symptoms - gritting teeth, wincing, localizing pain to right ankle  **A**llergies - none  **M**edications - none  **P**ast medical/surgical history - right ankle sprain (age 22)  **L**ast meal: 6 hours ago (croissant with nutella and orange juice)  **E**vents leading to incident: Was walking down the trail and was distracted by something up ahead and accidentally stepped into a hole where his foot got stuck. His right foot inverted/plantar flexed and he heard a low cracking noise, after which he felt a sharp pain in the medial and lateral aspects of his ankle. He has since been unable to walk, and notes that the joint feels a bit ‘loose.’ He has been sitting next to the trail x 1.5 hours. He does not have any other musculoskeletal injuries. Did not bring water, food, medical pack or extra layers. Cell phone fell out of his hand when the accident occurred and has been out of reach. He was unable to reach it due to pain.  Family history - mother: HTN, father: DM  If asked for review of systems:  Unable to walk on right ankle, localizes pain to medial and lateral aspects.  If asked about home environment/social history:  Lives with his wife in the city, did not tell her he was going on a hike because she was busy with work and he did not expect to be gone for long. |
| **Physical Examination** (initial impression) (primary and secondary assessment) | |
| **General** | Wearing hiking boots.  Alert, responsive.  Breathing a little faster than normal.  Is wincing in pain. |
| **HEENT** | Patent airway, lips are pale and dry, no lip/tongue swelling.  No obvious trauma to head.  PERRLA (*if have pen light to assess)* |
| **Neck** | Supple. |
| **Lungs** | Respiratory rate 20-25 breaths per minute, no audible abnormal air sounds. |
| **Cardiovascular** | Tachycardic, no murmurs/rubs/gallops. Radial pulses symmetric. |
| **Abdomen** | Soft, non-tender, non-distended.  No obvious trauma to abdomen. |
| **Neurological** | Alert, speaking in full sentences.  Sensation, cerebellar, tone, reflexes intact and symmetric. Unable to assess RLE motor exam due to pain, but otherwise grossly intact in remaining extremities.  No vertebral point tenderness. |
| **Skin** | No obvious bleeding.  Scattered superficial abrasions on bilateral hands.  No rash. |
| **Musculoskeletal** | Winces when you remove right boot. |
| **Psychiatric** | Upset but cooperative, non-combative. |

| **Instructor Notes - Changes and CASE Branch Points** | | |
| --- | --- | --- |
| **Intervention / Time point** | **Change in Case** | **Additional Information** |
| Hikers come across injured patient. | Begin case with patient on ground | Patient is yelling for help, and clearly in pain. |
| Hikers stop and assess for safety, prior to approaching the patient to offer help. |  |  |
| Hikers help move the patient away from the edge of the trail. | Patient experiences pain with any movement of ankle |  |
| Hikers divide into roles:  Hiker #1: Team lead  Hiker #2: Survey  Hiker #3: Helper who performs patient interventions  Hiker #4: Activates EMS, then helps Hiker #3 |  | If limited communication from team, patient may ask, “Who are you? Can you help me?” |
| Hikers complete initial assessment, primary and secondary assessment. | Patient is alert, irritable but consolable.  Is breathing a bit fast, with intact and symmetric peripheral pulses. | Patient is annoyed at the full body exam but is amicable after an explanation of reasoning. |
| Following examination of lower extremity, hikers create and apply a splint to the joint.  If controlled environment and scope of course allows, perform a short carry of the patient. | PE: No obvious bony/joint deformity. Dorsalis pedis and posterior tibial arteries palpable, sensation intact. Tender to palpation on R lateral aspect of ankle. Ambulation is not tolerated. | Patient yells out in pain when his right ankle is palpated, particularly on the medial and lateral aspect. He is unable to bear weight. |
| EMS greet group at trailhead.  Hikers give sign-out of pertinent information. |  |  |

**Ideal Scenario Flow**

- The simulation starts and the participants set out on a hike then promptly come across an injured hiker in distress.
- They STOP and assess the scene for safety prior to approaching the injured hiker, specifically noting a lack of environmental or human dangerous situations.
- They are able to calm the young man by assuring him that they are there to help.
- They assign team roles (including team leader, survey, caller for help, and provider of patient care activities).
- They perform an initial patient assessment and continuously reassess along the way. They acknowledge that he likely has an unstable ankle fracture that will require splinting and repeated neurovascular checks.
- They obtain vitals, history, and perform initial assessment of the patient.
- Acknowledge that the patient has an injury to his right ankle, but acknowledge the potential for other MSK injury and head trauma so specifically incorporate a full assessment, ie: examine his head/neck/back for injury in addition to a concussion assessment (all will be negative).
- Carefully remove the boot to evaluate the patient's ankle. Evaluate for bony deformity/dislocation. Instructor advises students that the ankle appears swollen and deformed. Patient has a moulage of a large bruise.
- Assess pedal pulses/circulation. Assess neurologic status.
- Apply an improvised splint with whatever is available, including something hard and supportive, with ample padding (ie: SAM splint, sticks, clothing as padding, rope or tape to secure).
- Hikers will discuss evacuation options that are non-weight bearing including litters and carries (ie: improvised litter, wheelbarrow carry).
- They discuss evacuation options and since the patient cannot bear weight on his ankle,
- If possible, proceed with a simple, assisted evacuation to the trailhead to meet with EMS. The evacuation should not make the injury worse or put others at risk for injury. If not possible based on the scope of the SIM, students should discuss this option without performing it.
- They pretend to activate EMS by calling 911 on their cell phones once they get service (the facilitator will tell them when they are in service range and reminds them not to actually call 911) and the scenario ends when EMS arrives and the participants give a thorough patient sign-out.

**Anticipated Management Mistakes**

- Failure to obtain pertinent history and physical and recognize the patient’s condition. If the learner does not obtain the salient points of the history and physical that suggest ankle injury, then the simulated patient actor can volunteer this information to the learner by exaggerating the symptoms. Likewise, the hike leader/facilitator can make a suggestion (ie: “why is he moaning in pain, he doesn’t seem to be able to bear weight on it, etc.”)
- Failure to stabilize the injured ankle well with a well-padded, rigid, improvised splint. If the learners attempt to extract the patient prior to splinting, the simulated patient actor can refuse to walk due to pain. The hike leader/facilitator can prompt (“I think I have a SAM splint somewhere,” “I have hiking poles, would those help?” or “look at all those rigid sticks on the ground.”). Additionally, facilitators should confirm immobilization of the injury above and below the joint.
- Failure to call for help. If this occurs, the facilitator eventually can provide the cell phone or indicate that cell service is working by getting phone alerts.
- Failure to extract patient. This is a step that will change, depending on the context. If they don’t have those skills, then upon splinting the ankle the team will perform a very short carry if on stable ground. If not, should verbalize “we are doing a carry.” The simulated patient will be alerted ahead of time to which scenario to expect. If the learners try to carry out the patient without knowing how to, the simulated patient will inform the learners that the case is over.
- Discussion Point: Access to commercial medical equipment (SAM splints, ACE wraps) may not always be available in the wilderness, facilitators should emphasize the role of improvisation and pointers on how to use available materials to create rigid splints.
