# Supplementary material for: Implementation of a Novel Wilderness Medicine Simulation Course for Medical Students
Source: MedEdPORTAL. 2025 Jun 9;21:11526. doi: 10.15766/mep_2374-8265.11526 (PMC12146433; doi:10.15766/mep_2374-8265.11526)
Supplement: Supplementary file 1 — WM Case 1.docxWM Case 2.docxWM Case 3.docxWM Case 4.docxWM Case 5.docxPre- and Postsurvey.docxPrebriefing and Learner Training Materials.docxCommon Curriculum Clinical Objectives.docx [file mep_2374-8265.11526-s001.zip › C. WM Case 3.docx]

This appendix is to be used to guide the flow of each simulated case scenario. The “instructor notes – changes and case branch points” and “ideal scenario flow” sections provide especially detailed instructions on how the simulation actors and facilitators should respond to different actions by the learners. Key learning objectives are listed on the first page.

| **Appendix C: Case 3: Traumatic Bleeding Control**  **SIMULATION CASE TITLE:** *Traumatic Arterial Bleeding Control: Wilderness Medicine Emergency Simulation for Medical Students*  **AUTHORS:** Kira Palazzo, Sophia Redpath, Katherine Sprengel | |
| --- | --- |
| **PATIENT NAME:** Samantha (Sam) Brown  **PATIENT AGE:** 24 years old  **CHIEF COMPLAINT: “I fell and my arm just keeps bleeding!”** | |
|  | |
| **Brief narrative description of case** | You are with a group hiking up a trail when you come across a lone adult female who appears to be in pain and is shivering. She is lying down next to the trail, is holding her right arm, and is alert. She calls out to you to help her.  The anticipated interventions of the responders include: (1) assessing the scene for safety; (2) assessing the patient, recognizing and initiating treatment for upper extremity arterial bleed including placement of tourniquet; (3) activating the emergency medical system (EMS) and transporting the patient to safety; and (4) using effective interpersonal communication.  Anticipated interventions include ongoing assessment of ABCs, field stabilization of arterial bleed, effective communication, and activation of EMS with safe transport to a higher level of care.  The patient stabilizes after bleeding is controlled and can walk with assistance. |
| **Primary Learning Objectives** | By the end of this activity, learners will be able to:   1. Assess the scene for safety prior to responding to an injured or incapacitated patient. 2. Assess the patient, recognize an injured hiker with arterial hemorrhage of right arm and provide effective initial stabilization and management. 3. Effectively activate the Emergency Medical System (EMS) in a remote wilderness setting and safely transport and sign out the patient. 4. Demonstrate effective teamwork and communication skills while managing an emergency in a remote setting. |
| **Critical Actions** | ***Crucial:***   1. Assess and constantly reassess the scene for safety 2. Primary, secondary survey 3. Recognize, treat bleed (use PPE as available, apply direct pressure with gauze for 10 minutes, if bleeding still uncontrolled then apply tourniquet) 4. Activate EMS as soon as cell service is available 5. Assign clear team roles and responsibilities 6. Communicate effectively as a team, including using directed, closed-loop communication 7. Give comprehensive patient sign-out to EMS |
| **Learner Preparation** | Learners will be briefed prior to the exercise regarding the availability of simulated cellular service, the availability of simulated EMS teams, and how to access and activate these systems in the simulated outdoor wilderness environment |

| **Initial Presentation** | |
| --- | --- |
| **Initial vital signs** | HR: 140 RR: 20 Temp: cool to touch |
| **Overall Appearance** | Sam is lying alone next to the trail, shivering, whimpering and holding her right arm. She is notably upset. Has cold, pale fingers on her right hand. |
| **Actors and roles in the room at case start** | Group of 4 hikers (medical students) respond to injured patient on trail and divide into roles:  Hiker #1: Team lead  Hiker #2: Survey  Hiker #3: Helper who performs patient interventions (ie: assesses vital signs, stops bleeding, places tourniquet, helps evacuate patient)  Hiker #4: Activates EMS, then helps Hiker #3.  Simulated injured patient: Full body manikin with arterial bleeding ability or bleeding-arm trainer; If manikin unavailable or if more practical in a wilderness setting, patient may be enacted by an instructor/helper.  Instructor #1: Simulation instructor who will also act as debriefer.  Instructor #2: If a 2^nd^ instructor is available, cast them as EMS dispatch on the phone and/or EMS provider that arrives at the scene. |
| **HPI** | Instructor #1 volunteer vignette:  Sam is a 24-year-old female who was out hiking. She is a realtor, but enjoys going hiking on her days off and is hiking alone. She is found alone, cold and injured lying in the middle of the trail.  Simulated injured patient: when asked about leading events (SAMPLE):  **S**igns/symptoms - shivering, dizzy, tearful, localizing pain to right arm  **A**llergies - none  **M**edications - none  **P**ast medical / surgical history - previously healthy, no prior surgeries  **L**ast meal: 4 hours ago (scrambled eggs and toast with avocado)  **E**vents leading to incident: Was walking down the trail and stepped onto a rock that was not stable, causing her to fall into a nearby tree that had a sharp branch sticking out. She tried to break her fall by reaching out, but in doing so, the stick pierced her right forearm and she has been unable to stop the bleeding. She notes that it has been bleeding profusely, and seems to pulse. She has been sitting on the trail for 5-10 minutes. She does not have any musculoskeletal injuries. Did not bring water, food, medical pack or extra layers.  Family history - none  If asked for review of systems:  Feels cold, slightly woozy. Cannot recall the last time she urinated and does not feel the urge now. Cannot feel right fingers as they have gone numb. Unable to stand due to dizziness, and is squeezing her right arm to try to stop the bleeding.  If asked about home environment/social history:  Is staying with friends who live closer to hiking trails. She does not recall their phone numbers by heart. |
| **Physical Examination** (initial impression) (primary and secondary assessment) | |
| **General** | Wearing dry clothing and hiking boots.  Alert, responsive.  Breathing a little faster than normal.  Is pale and is shivering. |
| **HEENT** | Patent airway, lips are pale and dry, no lip/tongue swelling.  No obvious trauma to head.  PERRLA (*if have pen light to assess)* |
| **Neck** | Supple. |
| **Lungs** | Respiratory rate 20-25 breaths per minute, no audible abnormal air sounds. |
| **Cardiovascular** | Tachycardic, no murmurs/rubs/gallops. Radial pulses asymmetric (decreased on L, 1+) |
| **Abdomen** | Soft, non-tender, non-distended.  No obvious trauma to abdomen. |
| **Neurological** | Alert, speaking in full sentences.  Sensation, motor, cerebellar, tone, reflexes intact and symmetric.  No vertebral point tenderness. |
| **Skin** | Cold, pale skin. Shivering. Capillary refill 3-4 seconds (slow).  Obvious bleeding to R forearm, appears to be arterial in nature.  Scattered superficial abrasions on bilateral hands.  No rash. |
| **Musculoskeletal** | Normal. |
| **Psychiatric** | Upset (crying) but cooperative, non-combative. |

| **Instructor Notes - Changes and CASE Branch Points** | | |
| --- | --- | --- |
| **Intervention / Time point** | **Change in Case** | **Additional Information** |
| Hikers come across injured Sam. |  | Sam is yelling for help, crying, and shivering. |
| Hikers stop and assess for safety, prior to approaching patient to offer help. |  | Sam is calmed by their presence, stops crying, but continues to shiver. |
| Hikers help move patient out of trail. |  | Sam yells out in pain that her right arm is “killing her” and she cannot release her arm. |
| Hikers divide into roles:  Hiker #1: Team lead  Hiker #2: Survey  Hiker #3: Helper who performs patient interventions  Hiker #4: Activates EMS, then helps Hiker #3 |  | Sam yells: “Can you help me please? I’m bleeding really badly here.” |
| Hikers complete ALS initial assessment, primary and secondary assessment. | Patient is alert, irritable but consolable.  Is breathing a bit fast, with intact and asymmetric radial pulses but otherwise normal pulses to BLE and LUE.  Skin is pale and cool to touch distal to injury with delayed capillary refill. | When asked what happened, Sam tells them she tripped about 5 minutes ago and her arm was impaled on a large, very sharp stick. She pulled the stick out and has not stopped bleeding. She is in a lot of pain. |
| Recognize shivering, pallor, decreased capillary refill, dizziness with standing as signs of volume depletion via blood loss. |  | Sam reports that she has been too dizzy to stand, and indicates that she feels really cold despite the warm weather. |
| Carefully remove patient’s hand from injury. The “injured arm” is a plastic manikin training arm with moulage of a deep, bleeding wound. | PE: Obvious wound to distal right upper extremity, appears relatively clean with no foreign body contamination. Pulsating bleeding coming from wound indicates arterial bleed. Decreased pulses on right arm No obvious bony/joint deformity. | Sam screams in pain when her right arm is inspected. She is initially resistant to having team examine her, but eventually agrees. |
| Hikers continue to assess vitals and monitor bleeding control. Administer rehydration via a water bottle from their pack, and give extra layers if she is still cold. | Radial pulse is absent while tourniquet is applied. | Sam is able to walk out with support from the hiking team. |
| Hikers assist Sam in walking to trailhead where EMS greet group. Hikers give sign-out of pertinent information. |  | Sam cries in relief at the sight of EMS, thanking the hikers as she is taken to hospital. |

**Ideal Scenario Flow**

- The simulation starts and the participants set out on a hike then promptly come across an injured hiker in distress.
- They STOP and assess the scene for safety prior to approaching the injured hiker, specifically noting a lack of environmental or human dangerous situations.
- They are able to calm the young woman by assuring her that they are there to help.
- They assign team roles (including team leader, survey, caller for help, and provider of patient care activities).
- They perform an initial patient assessment and continuously reassess along the way. Acknowledge that the patient has an injury on her right arm, so specifically incorporate a full assessment, ie: assess all clothing, examine her head/neck/back for injury. They acknowledge that she likely has an arterial injury that requires immediate intervention.
- One team member immediately puts on PPE and then applies direct pressure to the wound using gauze
- Another team member obtains vitals, history, and performs initial assessment of the patient
- Evaluate for debris/contamination and remove as necessary. Assess upper extremity pulses/circulation. Assess neurologic status. Apply direct pressure to wound and hold for 10-15 minutes (simulated), apply tourniquet 5-10 cm proximal to wound if bleeding is not controlled.
  - Following a period of time when bleeding is not controlled, a tourniquet is placed proximal to the wound
- They discuss evacuation options, and after ensuring that the bleeding is well controlled and the patient’s vitals are stable, and proceed with a simple evacuation.
- They pretend to activate EMS by calling 911 on their cell phones once they get service (the facilitator will tell them when they are in service range and reminds them not to actually call 911) and the scenario ends when EMS arrives and the participants give a thorough patient sign-out.

**Anticipated Management Mistakes**

- Failure to obtain pertinent history and physical and recognize the patient’s condition. If the learner does not obtain the salient points of the history and physical that suggest an arterial bleed with volume depletion, then the simulated patient actor can volunteer this information to the learner by exaggerating the symptoms. Likewise, the hike leader/facilitator can make a suggestion (ie: “she looks like she’s shivering, the bleeding seems to still be pretty bad, why is he moaning in pain.”)
- Failure to monitor bleeding and apply tourniquet as needed: Once determined that it is safe to approach the patient and that the patient is alert and cooperative, the team must recognize that the bleeding is pulsatile and likely arterial and put on PPE before attempting to staunch the bleeding. This should be done before continuing the assessment and checking of vital signs. If the learners do not make the effort to apply direct pressure and reassess, then the patient should report that she is starting to feel faint. The hike leader/facilitator can intervene with a prompt (ie: “maybe you should try making a tourniquet from supplies in your backpack”)
- Attempt to remove tourniquet after placement:
- Failure to reassess vital signs: once the bleeding is controlled, team members should reassess vital signs, especially checking for signs of further volume depletion (increased cap refill time or increased HR) or altered mental status.
- Failure to call for help. If this occurs, the facilitator eventually can provide the cell phone or indicate that cell service is working by getting phone alerts.
- Failure to extract patient. This is a step that will change, depending on the context (i.e. the patient’s volume status). If the patient is alert and oriented, she can be helped to safety with assistance. If she has decreased mental status, then the team will need to inform EMS that they will need to carry her out. The simulated patient will be alerted ahead of time to which scenario to expect. If the learners try to carry out the patient without knowing how to, the simulated patient can insist on trying to walk using a cane or an arm as support.
- Discussion Point: Absent radial pulses with tourniquet application, conversation about removal of tourniquet. Improvising a tourniquet using a windlass (ie. a firm stick) to provide the force to adequately tighten the band (a piece of clothing, inflexible bandage, or strap) for hemostasis.
